# Telehealth, COVID-19 and Refugees and Migrants in Australia: Policy and Related Barriers and Opportunities for More Inclusive Health and Technology Systems

**DOI:** 10.34172/ijhpm.2021.31

**Published:** 2021-04-10

**Authors:** Ben O’Mara, Devaki Monani, Gemma Carey

**Affiliations:** ^1^Department of Media and Communication, Faculty of Health, Arts and Design, Swinburne University, Melbourne, VIC, Australia.; ^2^School of Social Work, College of Health and Human Sciences, Charles Darwin University, Casuarina, NT, Australia.; ^3^Centre for Social Impact, University of New South Wales, Sydney, NSW, Australia.

## Introduction

 People from refugee and migrant communities of culturally and linguistically diverse backgrounds in Australia, including asylum seekers, the elderly and others who are particularly vulnerable, have experienced major social, economic, physical and mental health issues associated with the coronavirus disease 2019 (COVID-19) pandemic. Recently, the Australian government made changes to Medicare legislation,^1^ the country’s universal health insurance scheme,^2^ for subsidising access to medical care and advice via telehealth as a response to the pandemic. Evidence suggests, however, that many refugee and migrants have not been able to experience the benefits of the changes, excluding them from an important source of medical care and information about virus infection and other health issues. Furthermore, the changes have not addressed ongoing challenges in general access to telehealth and the need for greater culturally and linguistically appropriate healthcare in a multicultural Australia.^3^ In this viewpoint piece, we draw on the World Health Organization (WHO) Promoting the Health of Refugees and Migrants: Draft Global Action Plan 2019–2023^4^ to show that the recent changes to Medicare legislation for telehealth due to the pandemic fail to adequately support refugees and migrants but are a unique opportunity for learning how to work towards more culturally and linguistically inclusive health and technology systems. We contend that extending eligibility for Medicare, adding refugee nurses, doctors and similarly qualified healthcare providers to the My Health Records Act^5,6^ and similar potential policy changes are important to explore for better supporting refugees and migrants with telehealth during, and after, the pandemic. Research and evaluation are also necessary to better understand recent legislative changes and how to develop more culturally and linguistically inclusive healthcare using telehealth and in offline settings.

## Refugees and Migrants, COVID-19 and Health and Technology Policy in Australia

 Refugees and migrants have experienced major health issues associated with COVID-19, issues that initial reporting suggests may have become worse due to barriers in accessing culturally and linguistically appropriate medical care and information.^5^ A lack of appropriate information and concern about being reported to government and VISA cancellation, detention or deportation have prevented many refugees from being tested for COVID-19.^5,7^ There was an increase in reports of refugees with a mental health condition posing a serious risk to their safety, including suicide and self-harm.^5^ Medicare has not been available to refugees who have temporary VISAs,^8^ meaning they have not been able to pay for counselling, medication and other health support, increasing stress and the risk of health problems.^5,9-13^

 Many migrants face similar challenges as refugees with COVID-19. Finding culturally and linguistically appropriate information and support for dealing with stress, depression and other mental health conditions during the pandemic has been very challenging.^7^ International students have reported experiences of racism and difficulty finding appropriate support for managing the pressures associated with study and financial instability.^14^ Elderly migrants have been more vulnerable to health issues^7^ and can lose the ability to use English and prefer communicating in their first language,^15^ making understanding information about COVID-19 difficult, increasing their risk of infection.

 In March of 2020, the Australian Federal Government made temporary changes to the Medicare Benefits Schedule to help people use telehealth for accessing medical care and advice while also reducing the risk of infection from COVID-19.^1^ The changes included the adding of videoconferencing and telephone items for a range of services, including standard general practitioner and Nurse practitioner attendance, health assessment for people of Aboriginal or Torres Strait Islander descent, chronic disease management, pregnancy support counselling, eating disorders, mental health, dental and neurosurgeon, psychiatrist and other specialist attendance.^1^ As a result, a range of telehealth services became government subsidised for those with access to Medicare, and the ability to easily use and access information technology like video conferencing and the internet. Initial reporting suggests that the changes to Medicare for telehealth were associated with a major increase in its use by the general Australian population, and helped to reduce the risk of infection by allowing health professionals to provide a level of medical care without patients having to physically visit a clinic.^16^ Despite the promising uptake of telehealth and its potential for reducing infection, the legislative changes did not address the health issues and barriers facing many refugees and migrants (see Table).

**Table:** Summary ofLegislative and Related Barriers and Opportunities for Refugee and Migrant Use of Telehealth in Australia

**Barriers**	**Opportunities**
Medicare ineligibility for those on temporary VISAs	Extend and evaluate access to Medicare for temporary VISA holders so that they can access more affordable healthcare in general, including telehealth
My Health Records Act does not provide guidance for translation of information and culturally and linguistically appropriate medical care	Amend the My Health Records Act to recommend the use of appropriately qualified health professionals for refugees and migrants and accredited translators in telehealth
Ineligibility for government funded support for education, employment, disability and other social safety needs for those on temporary VISAs	Extend and evaluate access to government funded social safety net support for those on temporary VISAs to help address economic, education and other social determinants of health for improved access to telehealth
Isolation, trauma, stigma, a lack of social support, environmental barriers, low socioeconomic status and not enough culturally and linguistically appropriate health information and services	Add and evaluate Medicare items for refugees and migrants, including telehealth, to help develop more appropriate medical care and information and support greater investment in the health work force
Poor quality internet, a lack of access to affordable internet and relatively low levels of understanding and skills in using technology for health	Research what may help address the health, employment, education, training and other social safety needs of refugees and migrants, including the elderly, women and/or those with low levels of health literacy, education and language proficiency for accessing online and offline medical care and information

 Medicare ineligibility prevents many refugees and migrants on temporary VISAs from government funded support for telehealth. Government funded support for telehealth is only available through Medicare.^1^ Therefore, people with temporary VISAs cannot access telehealth at a subsidised cost using the temporary Medicare items for videoconferencing and telephones, including general health assessments, dental care and counselling and other forms of support for trauma, stress and other mental health issues, services that may be particularly beneficial for refugees and migrants.

 The problems associated with limited access to Medicare subsidised Telehealth are reinforced by the My Health Record Act (2012). The Act is a major piece of health and technology legislation in Australia that helps to govern the use and access of health information online, including telehealth, and demonstrates significant gaps in how it addresses the needs of refugees and migrants. Currently, the Act does not include guidance on the need for appropriately qualified health professionals who can help provide more culturally and linguistically inclusive medical advice and care to refugees and migrants using telehealth, including addressing issues related to privacy and confidentiality. Evidence suggests that cultural beliefs relating to the sharing of health information differ for many refugees and migrants. Similarly, the My Health Records Guidelines do not recommend the use of accredited translators in telehealth, which could make the technology easier to access through use of preferred languages in medical appointments.

 Compounding the legislative barriers are other health system, cultural and technological limitations in the use of telehealth for refugees and migrants in Australia (see Table). Clinicians and patients still experience problems with telehealth sessions, including difficulties with obtaining prescriptions and pathology results, the additional burden for complex care, and the inability to be physically examined.^16^ Those who are elderly, experience disability and/or suffer from diabetes, anxiety, drug dependency and other chronic conditions generally find it difficult to access appropriate health services for their needs. Isolation, trauma, stigma, a lack of social support, environmental barriers and not enough culturally and linguistically appropriate information and services can all severely limit the use of telehealth by refugees and migrants.^17-21^ Research also shows that online platforms are generally not easy to access and use for many refugees and migrants in Australia, with poor quality internet, a lack of access to affordable technology and relatively low levels of understanding and skills in using technology for some groups, including new and emerging platforms.^15,17^

 Legislative and other barriers too telehealth are deeply problematic and avoidable. The barriers contribute to an increased the risk of health and safety issues associated with COVID-19 for refugees and migrants on temporary Visas, and the broader and multicultural population of Australia as a whole.

## WHO Guidelines and Opportunities for More Inclusive Telehealth Policy Change

 The barriers to Medicare and telehealth suggest important areas of focus for future legislative work that could improve support for refugees and migrants with telehealth, namely the Medicare and the My Health Record legislative systems (see Table). The government’s broader social safety net systems also show great potential for future legislative work that is highly relevant to more inclusive telehealth, as does targeted investment the Australian health workforce, research and evaluation.

 To help guide future legislative and policy work on telehealth, the WHO’s recommendations for supporting refugee and migrant health are pertinent for developing options for action that may be most beneficial. Engaging with WHO recommendations on refugees and migrants is also in line with the Australian government’s broader approach to collaboration with WHO for addressing the global spread of COVID-19, as well as considering WHO strategy and decision-making for Australian health policy.^22^

 The main WHO recommendations, from WHO’s Promoting the Health of Refugees and Migrants: Draft Global Action Plan 2019–2023, can be summarised as the need to better promote refugee and migrant health across national agendas, public health interventions, essential healthcare, employment and other social determinants of health, health monitoring and information systems. The strategy also recommends the use of evidence-based health communication with refugees and migrants, and challenging misperceptions about migrant and refugee health in health systems and society more broadly. Changes to Medicare, the My Health Record Act and investments in related health workforce and research activities for telehealth can build on the WHO recommendations by generally working towards the development of an Australian health system that is much more capable of meeting the linguistic, cultural, social, financial and physical and psychological needs of refugees and migrants.

 The first area of legislative work, and possibly most important, is access to Medicare (see Table). There is a need to extend access to Medicare for all refugees and migrants who are temporary VISA holders in Australia so that they can access more affordable healthcare in general,^5,9^ and to use subsidised telehealth. Additional Medicare items may also help. By adding specific videoconferencing and telephone services Medicare items for refugees and migrants, it can improve access to support for their specific needs, such as trauma and related mental health conditions, dental health,^11^ sexual and reproductive health and immunisation,^10,23^ and help reduce the likelihood of some refugee health conditions becoming chronic.^13^ With access to government subsidised telehealth and specific Medicare items, many refugee and migrants on temporary VISAs are likely to have another option for meeting their physical and psychological needs.^12^ Changing Medicare may help to better mainstream refugee and migrant health by improving their visibility in the Australian health system, and the system’s ability to provide more culturally and linguistically appropriate interventions in the short and long term, including targeted support for the elderly and others facing major barriers to telehealth and in need of face-to-face medical care. The change may also help to support investment in the health workforce for more appropriate service provision.

 In the second instance, there is potential to amend the My Health Records Act (2012) and its associated guidelines (see Table). Amending the act to address the need for appropriately qualified health professionals is likely to help provide more culturally and linguistically inclusive medical advice and care to refugees and migrants. Similarly, including recommendations in the My Health Records Guidelines for use of accredited translators in telehealth may make it easier to access through use of preferred languages in medical appointments. With an Act and its guidelines more sensitive to the needs of refugees and migrants, it can support greater capacity for quality essential healthcare, more culturally and linguistically appropriate interventions, improved evidence-based communication and a strengthened health information system.

 The third area of potential work is to make changes beyond health and technology legislation, including addressing issues with social safety nets, and the need for research and evaluation (see Table). Changes that reduce legislative barriers to government support for employment, education, housing, training and language programs could play a major role in increasing the amount and diversity of refugees and migrants able to use the basic technological functions of telehealth. Extending access to government support for those on temporary VISAs to address issues with unemployment and language support programs,^24^ for example, may help more refugees and migrants to access the funds and develop the skills necessary telehealth. Such changes would also improve the Australian governments’ ability to address the social determinants of health and support evidence-based communication.^4^ The development of a national strategy for refugee and migrant health in Australia could also provide important guidance for the implementation of any changes and related activities. Currently, there is no national strategy for refugee and migrant health.

 Finally, research and evaluation need to establish the degree to which refugees and migrants use telehealth and other forms of information technology for health communication online (see Table). To help better understand refugee and migrant experiences with telehealth, future research needs to build on relevant past studies in Australia. There are only a small number of past studies into telehealth, and while they have major limitations with regards to generalisability, they do provide useful areas of focus. Firstly, national research must be conducted with a greater diversity within and across many refugee and migrant communities to improve the evidence base. The research must focus on the ability to, and preferences for, use of information technology when communicating on telehealth, as well as any barriers that may be experienced. In the second instance and in line with previous telehealth research, there is also an opportunity to determine the effectiveness of accessing an interpreter via videoconference for medical appointments at a national level, a form of telehealth that evidence in the state of Victoria suggests is promising with refugees and migrants.^20^ Quantitative, survey-based research conducted during out-patient clinical consultations should be considered as a methodological approach.

 Similarly, qualitative research shows potential for improving the evidence base and complementing quantitative research. Past qualitative studies suggest refugees and migrants of different age groups and with different levels of educational background and English language ability use the internet and other online platforms in meaningful and positive ways for their health, including accessing health information in languages other than English.^24^ However, barriers to information technology and health information persist, especially for older people, women with family responsibilities and those with relatively lower levels of literacy and skills in the use of information technology. In some communities, there are also cultural preferences relating to the importance of trust and face-to-face communication for health. The barriers and opportunities to online and face-to-face health information and medical care must be further investigated. Participatory techniques with a diverse range of people from refugee and migrant communities have the potential to better reach many refugees and migrants given ongoing issues with engagement in the health system and the English language barriers of many existing national health surveys.^24^

 Evaluation of the recent policy changes to Medicare for telehealth is also necessary. Evaluation needs to increase understanding of impact from the changes, at a national level, with refugees and migrants. With a better understanding of impacts from the changes, it is possible to contribute insight into what future policy changes, research and practical work may be effective for supporting refugee and migrants to access telehealth. The Australian government’s national standdown and evaluation measures for COVID-19,^22^ and participation in WHO’s Independent Panel for Pandemic Preparedness and Response to evaluate the world’s response into COVID-19, are appropriate mechanisms for developing and implementing evaluation of the Medicare and Telehealth changes in line with public health and health policy processes, and for generating understanding of their impacts on refugees and migrants, and what may help with future waves of the virus.

## Conclusion

 Policy-makers in Australia and across the world have shown they are able to respond swiftly in order to try and prevent infection and harm from COVID-19. Importantly, legislative changes supporting innovative approaches, like the use of government subsidised telehealth, have contributed to the declining rates of infection in Australia. The speed of policy action shows what is possible when dealing with emergencies like the current pandemic, including support for the health and well-being of migrants. However, the pandemic has also shown how changes to Medicare have not addressed major barriers to Australia’s universal health insurance scheme. The barriers contribute to an increased risk of health problems and other issues for refugees and migrants in Australia.

 Now there is a rare opportunity to apply learning from past work and the recent changes to Medicare legislation to help strengthen the healthcare system, and the health and well-being of refugees and migrants across Australia. Future policy development and research activities are pivotal for establishing how Medicare and technology legislation changes may best address the needs of refugees and migrants, support investment in the professional development of the healthcare workforce, and a national approach for more culturally and linguistically inclusive telehealth. With more inclusive health systems using telehealth and other technology, we can work towards more people across Australia feeling safe, and supported, as we continue to deal with the impacts of COVID-19.

## Acknowledgements

 This article was made possible through an Adjunct Research Fellow position at the Department of Media and Communication, Faculty of Health, Arts and Design, Swinburne University. The research built on the findings of previous postdoctoral research at Victoria University into the views of refugee and migrant communities from Vietnamese, Sudanese and Samoan backgrounds in Melbourne’s west, and their experiences with information technology for health and well-being needs.

## Ethical issues

 Not applicable.

## Competing interests

 Authors declare that they have no competing interests.

## Authors’ contributions

 Conception and design: BO, DM, GC. Acquisition of data: BO. Analysis and interpretation of data: BO, DM, GC. Drafting of the manuscript: BO, DM, GC. Critical revision of the manuscript for important intellectual content: DM, GC.
